# Social affective context reveals altered network dynamics in schizophrenia patients

**DOI:** 10.1038/s41398-017-0055-9

**Published:** 2018-01-31

**Authors:** Talma Hendler, Gal Raz, Solnik Shimrit, Yael Jacob, Tamar Lin, Leor Roseman, Wahid Madah Wahid, Ilana Kremer, Marina Kupchik, Moshe Kotler, Maya Bleich-Cohen

**Affiliations:** 1 Functional Brain Center, Tel Aviv Sourasky Medical Center, Tel Aviv, Israel; 2 Sackler Faculty of Medicine, Tel Aviv University, Tel Aviv, Israel; 3 School of Psychological Sciences, Tel Aviv University, Tel Aviv, Israel; 4 Sagol School of Neurosceince, Tel Aviv University, Tel Aviv, Israel; 5 Film and Television Department, Tel Aviv University, Tel Aviv, Israel; 6 Mazra Mental Health Center, Acre, Israel; 7 Beer Yaakov Mental Health Center, Beer Yaakov, Israel

## Abstract

Impairments in social cognition and interactions are core psychopathologies in schizophrenia, often manifesting as an inability to appropriately relate to the intentions and feelings of others. Neuroimaging has helped to demarcate the dynamics of two distinct functional connectivity circuits underlying the social-affective processes related to mentalization (known as Theory of Mind, ToM) and somatic-affiliation (known as Embodied Simulation, ES). While evidence points to abnormal activation patterns within these networks among those suffering from schizophrenia, it is yet unclear however, if these patients exhibit this abnormal functional connectivity in the context of social-affective experiences. The current fMRI study, investigated functional connectivity dynamics within ToM and ES networks as subjects experienced evolving cinematic portrayals of fear. During scanning, schizophrenia patients and healthy controls passively watched a cinematic scene in which a mother and her son face various threatening events. Participants then provided a continuous and retrospective report of their fear intensity during a second viewing outside the scanner. Using network cohesion index (NCI) analysis, we examined modulations of ES-related and ToM-related functional connectivity dynamics and their relation to symptom severity and the continuous emotional ratings of the induced cinematic fear. Compared to patients, healthy controls showed higher ES-NCI and marginally lower ToM-NCI during emotional peaks. Cross-correlation analysis revealed an intriguing dynamic between NCI and the inter-group difference of reported fear. Schizophrenia patients rated their fear as *lower* relative to healthy controls, shortly after exhibiting *lower* ES connectivity. This increased difference in rating was also followed by *higher* ToM connectivity among schizophrenia patients. The clinical relevance of these findings is further highlighted by the following two results: (a) ToM-NCI was found to have a strong correlation with the severity of general symptoms during one of the two main emotional peaks (Spearman *R* = 0.77); and (b) *k*-mean clustering demonstrated that the networks’ NCI dynamic during the social-affective context reliably differentiated between patients and controls. Together, these findings point to a possible neural marker for abnormal social-affective processing in schizophrenia, manifested as the disturbed balance between two functional networks involved in social-affective affiliation. This in turn suggests that exaggerated mentalization over somatic-affiliative processing, in response to another’s’ distress may underlie social-affective deficits in schizophrenia.

## Introduction

Impairments in social cognition and interactions are core psychopathologies in schizophrenia and highly relevant to the patients’ functional outcome^1^. More specifically, schizophrenia patients show deficits in understanding the intentions of others and establishing trusting, intimate relationships—crucial components for leading a viable and fulfilling social life. Moreover, such social-cognitive deficits often invoke an affective state of fear or aversion of others (i.e. paranoia or avoidance of social interactions), leading to the exacerbation of the patient’s emotional well-being. Such deficits manifest in various cognitive-affective domains, including attention allocation, recognition and interpretation of affective social contents, and social motivation^2^. Indeed, sibling studies point to a hereditary basis for impairments in social cognition and interaction among those suffering from schizophrenia^3,4^. Thus, identifying the neural underpinnings of such social-affective deficits in schizophrenia could provide markers for vulnerability and/or clinical status, as well as targets for early interventions or preventive treatments for siblings.

Two main processes have been suggested to underlie social cognition in humans; the process of mentalization, also known as *Theory of mind* (ToM), refers to the ability to attribute various mental states to our own, as well as to others’, beliefs, intentions, goals and behavior; somatic-affiliation, also known as *embodied simulation*(ES) or affective resonance, denotes the internalization of another’s visceral and affective state by vicariously creating mental simulations of such states (for a review see ref. ^5^). Neuroimaging and lesion studies have both indicated that these two modes of social affective processes indeed rely on different neural circuits, with ToM implicating a medial prefrontal—temporo-parietal network, and ES associated with a neural circuit that includes the anterior insula and the anterior cingulate cortex (ACC)^5^.

Importantly, behavioral and neuroimaging studies on schizophrenia patients and individuals at high risk for schizophrenia demonstrate impairments in these processes^6–10^. Imaging studies have revealed that several nodes within the ToM network are profoundly altered in schizophrenia^6,9–11^. In addition, schizophrenia patients also show abnormal structure^12^, function^11,13,14^, and connectivity^15^ between major ES nodes, including the anterior insula and anterior cingulate cortex (ACC). However, studies that reported abnormalities in ToM-related and ES-related nodes used static stimuli lacking any social-affective context, and thus weakening the ecological validity of these findings with regards to social cognition.

The current study provides a unique approach by attempting to probe schizophrenia-related deficits in the functional dynamics of networks that are involved in social affective processing; namely ES and ToM. The neural dynamics of these processes were rendered using a naturalistic emotional cinematic experience that depicted a threatening scene in which two characters (a mother and son) face the development of a dreadful situation. The approach for portraying the functional dynamics of the networks chosen a priori was based on our previous work showing a continuous measurement of within-network and between-network cohesion (termed network cohesion index, NCI)^16^. Using this approach in a previous study we successfully delineated the dynamics between ES and ToM networks, among a group of healthy participants, while they passively watched cinematic scenes that induced feelings of sadness. Specifically, we found that the NCI of these networks was related to an individual’s personal tendency to experience distress during the emotional peaks of the cinematic experience, as illustrated by an empathy trait questionnaire^17^. Especially intriguing was the observed dissociation between the NCI patterns of these two networks during peaks of sadness throughout the threatening scene; while the ToM network showed reduced NCI, the ES network exhibited increased NCI. Based on these studies, and taking into accounting the known difficulties in social cognition and interactions, we anticipate that the schizophrenia patients will provide a blunted continues report of their emotional experience during peak moments of threat in the film clip relative to healthy controls. In addition, in line with the previously described abnormalities found among schizophrenia patients in the major nodes of ToM and ES networks, we also expect to find abnormal NCI dynamics between the ES and ToM networks relative to healthy controls. Lastly, we maintain that the disrupted network cohesion patterns in schizophrenia will be associated with abnormal modulations in their reported emotional intensity, as well as symptom severity.

## Materials and methods

### Subjects

The study groups included 30 inpatients (13 women; mean age 25, range: 18–40 years) recruited by a senior psychiatrists at one of three Mental Health Centers: Beer Yaakov Mental Health Center, Mazra Mental Health Center and Lev-Hasharon Mental Health Center. Patients were diagnosed based on the structured clinical interview for DSM-IV Axis-I disorders, Patient Edition, SCID-I/P (First, 1994), while hospitalized. Those with major affective or neurological disorders, or drug-induced or alcohol-induced psychoses were not included. Medical and neurological illnesses were ruled out by physical and neurological examinations, routine laboratory investigations, reports from the patients’ family physicians and the hospital medical records. Severity of schizophrenia symptoms were assessed with the Positive and Negative Syndrome Scale (PANSS)^18^ (Table S1). Twenty-three healthy individuals without any known psychiatric or neurologic disorder (12 women; mean age 26, 22–39 years) were recruited through public advertisement (Table S1). Patients and healthy controls were matched for age and gender. All participants were native Hebrew speakers with adequate language comprehension. After drop-out, due to motion artifacts (see below), our final sample included 27 patients (10 women; mean age 25, range: 18–40 years) and 22 healthy controls (11 female, mean age: 26, range: 22–39). There is no standard way to perform power analyses for non-parametric analyses, such as those that are employed in this work. The estimated power of a corresponding parametric *t*-test with the same number of participants as in the selected sample is 0.77 for ToM NCI, and 0.99 for ES NCI. These values were estimated based on data from the time window of the highest mean difference between these the group NCIs (following ref. ^19^).

The study was approved by the Institutional Review Boards in each of the mental health centers. All participants gave written informed consent after receiving a full explanation of the study protocol.

### Task

The cinematic stimulus was selected due to its high ecological validity and the efficiency of movies as inducers of emotion^20^. Participants passively watched a clip from the movie “The Ring 2” (8:15 min) with Hebrew subtitles while in the scanner. The clip included two dramatic emotional peaks: a mother looking for her son while experiencing a frightful delirium in the amusement park restroom (~2 min), and a deer attacking the mother and son (2 min). Participants passively viewed a blank screen for 1 min before and after the film was presented.

## Continuous fear rating

Due to technical issues valid post-scanning continuous fear rating were obtained from 24 schizophrenia patients (9 females, mean age: 27.2, range: 19–40) and only 10 of the healthy controls (1 female, mean age: 26, range: 22–39). To improve the estimation of the rating pattern of the healthy population, we included data from 77 additional healthy volunteers. Thus, in total, the group included 87 healthy controls (33 females, mean age: 25.6, range: 20–40). It should be noted that the median ratings of the smaller and later expanded healthy control groups were highly correlated (Spearman *R* = 0.98, *p* < 5 × 10^−102^).

In the post-scan session, the participants watched the movie clip for a second time while continuously and retrospectively rating the fear intensity they recalled feeling during the first viewing in the scanner. The rating was performed retrospectively to avoid the confounding effect of rating while being in the scanner for the first time. Fear was the targeted emotion because it can be considered an “empathic emotion” inducer^21,22^, since a threat is being posed to a cinematic character rather than to the subject themselves. The rating was sampled at 10 Hz using in-house software^16^. The participants used a vertical scale indicating 7 levels of fear—from neutral to very deep (each containing 3° of shift; 21° in total).

### fMRI data acquisition and preprocessing

fMRI scanning was performed using a GE 3 T Signa Excite echo speed scanner with an eight-channel head coil located at the Tel-Aviv Sourasky Medical Center. Structural scans included a T1-weighted 3D axial spoiled gradient echo (SPGR) pulse sequence (TR/TE = 8.94/3.48 ms, slice thickness = 1 mm, flip angle = 13°, pixel size = 1 mm, FOV = 256 × 256 mm). Functional whole-brain scans were performed in interleaved order with a T2*-weighted gradient echo planar imaging pulse sequence (time repetition [TR]/TE = 3000/35 ms, flip angle = 90°, pixel size = 1.56 mm, FOV = 200 × 200 mm, slice thickness = 3 mm, 39 slices per volume). Active noise canceling headphones (Optoacoustics) were used.

Brain Voyager QX 2.4 was used for preprocessing and co-registration of the standardized anatomical and functional data. Head motions were detected and corrected using trilinear and sinc interpolations respectively, applying rigid body transformations with three translation and three rotation parameters. The data were high-pass filtered at 0.008 Hz and spatial smoothed with a 6 mm FWHM kernel. To avoid the confounding effects of fluctuations in the whole-brain BOLD signal, for each TR, each voxel was scaled by the global mean at that time point. Due to head movements (deviations higher than 1.5°/1.5 mm relative to the reference) three patients and one healthy control were excluded from the study.

Finally, we trimmed the functional data to improve the specificity of our analysis. The onset of the cinematic stimulus after a minute of rest may evoke emotional response regardless of the content of the film. Since we were not interested in this psychological effect, we removed a segment of five TRs from the functional data that were collected during the film viewing. The size of this segment was set to five TRs to discard the hemodynamic response to the film onset, as this response typically lasts 12–16 s. A segment including the five first time points was removed from the rating as well so that these signals could be comparable.

### Defining networks of interest

**Fig. 1:**
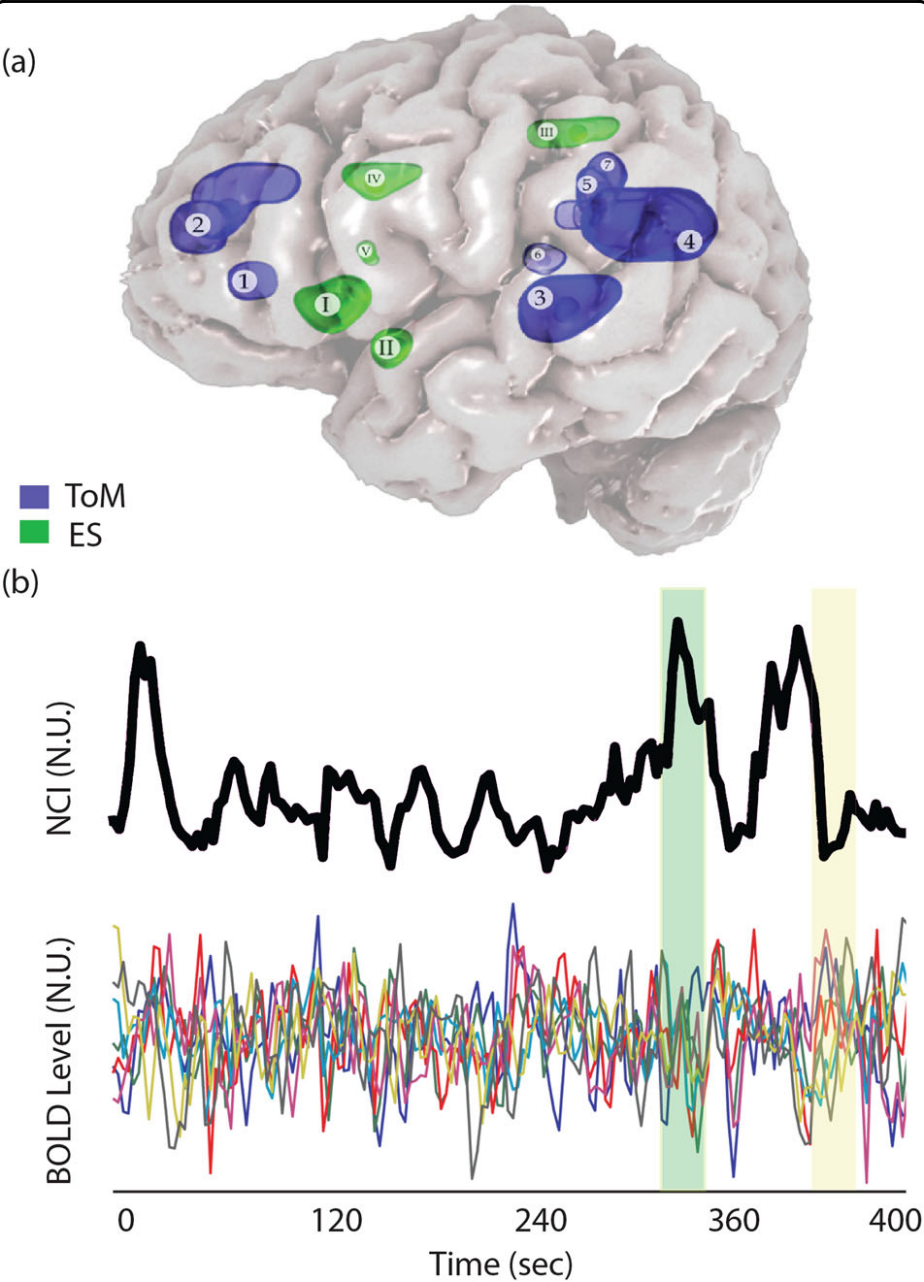
Network cohesion analysis approach **a** Anatomy of ES networks and ToM networks (based on Bzdok et al. ^24^; Lamm et al. ^23^). ToM network: (1) dorsal mPFC, (2) ventral mPFC, (3) left STS, (4) left TPJ, (5) precuneus (ventral), (6) right STS, and (7) right TPJ. ES network: (I) left AI/IFG, (II) left ventral AI, (III) precuneus (dorsal), (IV) ACC, and (V) right AI. **b** An illustration of the sensitivity of the network cohesion index to fluctuations in similarity of blood oxygen level dependent (BOLD) signals. Each of colored lines represents the BOLD time course of a different node in the ToM network (see the text for details) obtained from a representative subject. The upper black curve indicates the NCI computed for this network. NCI indicates the extent to which these signals are in synchrony in a certain time window. The green and the yellow rectangles mark intervals of increased and decreased NCI, respectively. Note that the homogeneity of the fluctuations of the signals differs between these intervals as indicted by the NCI

The anatomical definition of social cognition-related networks relied on two relevant meta-analyses of ES and ToM neuroimaging studies (Fig. 1a, Table S2). Both networks were used in a previous analysis of network cohesion applied to data collected during the viewing of two additional cinematic contents used in our lab^17^. The statistical maps were kindly provided by the authors of refs. ^23^ and ^24^, respectively.

The defined ES network was based on a meta-analysis of studies on vicarious pain experiences^23^ and included regions that were activated both during first-hand experiences of pain and while observing the pain of another. The defined ToM network was based on a meta-analysis of studies^24^ that involved taking the cognitive perspective of another’s mental state. Seven regions were selected from the meta-analytical statistical map, and based on further evidence from another study^25^ as being the only regions reliability activated across various ToM tasks.

### NCI analysis

All NCI analyses were performed using a designated Matlab code, which will be made available upon request. In short, NCI analysis (Fig. 1b) is a sliding-window method to estimate the functional connectivity dynamics between and within networks during continuous paradigms. NCI is a *t*-statistic based on the pairwise correlations of network nodes. It is sensitive to both the magnitude and variance of connectivity within the network, thus depicting cohesion between nodes. In previous works, NCI of specific networks of interest were found to covary with empathy and emotion reactivity indices^16,17,26–28^, pointing to their capacity to affect network dynamics.

To compute NCI, we first extracted the signal for each of the network nodes. The node’s signal was defined as the average signal of the voxel clusters reported by the relevant meta-analysis. For each network, time-window, and participant, the set of all pairwise Pearson correlations within the network was computed. The time-windows spanned over 30 s (10 TRs) according to previous evidence for the validity of using windows of this length^29^. We examined successive time windows with 90% overlap. NCI was computed as the *t*-statistic of a Student’s *t*-test with a null hypothesis of *µ*_R_ = 0, performed on the Fisher *z* transformed coefficients for each participant, network, and time-window. In this test, the *t*-statistic serves as a probe for the connectivity within the network, with elevated values when the mean correlation is high and variance is low.

### Correlation of NCI with fear rating group difference

We next investigated whether the difference between the fear ratings of schizophrenia patients and healthy controls were related to the corresponding inter-group difference in the connectivity of the ES and ToM networks. To examine the temporal relations between these parameters, we computed cross-correlations between NCI and the difference in rating indices, while displacing one time series relative to the other.

To fit the rating index with the sliding-window format used for NCI analysis, the data was first down-sampled to 30 s windows^16^. In each time-window we performed a non-parametric Wilcoxon test to compare the median rating of the schizophrenia and healthy control groups. A corresponding index for the inter-group NCI difference was similarly computed via a Wilcoxon test for the ES and ToM networks. The resulting *Z*-statistic time series were cross-correlated, as the NCI time-series were displaced relative to the rating difference series in a range of ±one time window with a single time point (one TR) lag (i.e., the NCI signal was shifted in up to ±10 TRs relative to the rating; see Fig. S1). This procedure yielded 21 NCI time courses (the non-shifted time series + 10 backward and forward shifted time courses) with 131 time-points each according to the following calculation (see Fig. S1 for visualization): The duration of the movie clip was 165 TRs. As mentioned above, five TRs were discarded from the data to avoid the film onset effect. For the time series of 160 TRs that were left after this trimming, we calculated 151 sliding NCI/rating time windows (10 TRs each). From this NCI time series of 151 TRs we then derived time series shifted in up to ±10 TRs. These shifted time series were generated in an identical size to allow proper comparison. Under these constrains, the maximal size of the shifted time series was 131 TRs.

Spearman’s correlation coefficients were computed for each of the lagged data sets; for 21 inter-group NCI and rating duplets with 131 time-points each. We determined the significance of the correlations by applying a bootstrapping procedure^30^ that included phase randomization for each Fourier component of the individual NCI time series and then the inverse Fourier transformation. To control for 21 comparisons for each of the two main hypotheses (ToM-NCI rating and ES-NCI rating associations), we applied false discovery rate (FDR) for independent or positively dependent tests^31^.

### Comparing NCI between groups

We further examined whether significant NCI differences between schizophrenia patients and healthy controls can be found at specific time-windows during the movie.

To compare network connectivity between the groups, a two-sided Wilcoxon test was performed on the two distributions of individual NCI values for each of the networks. This test was repeated for each of the time-windows. To control for multiple comparisons, FDR correction^32^ was applied considering the total number of comparisons for each network (131 comparisons).

We further examined whether differences between the groups are specifically related to the network connectivity or could also be found when comparing the time series of the BOLD amplitudes of these networks. Thus, we averaged the *Z*-scored signals obtained for all of the network nodes, averaged the signals again using a sliding windows of 10 TRs and compared the resulting time series between the groups using a two-sided Wilcoxon test.

### Correlating NCIs with schizophrenia symptoms

We next examined the co-variation between the network connectivity indices and the severity of schizophrenia symptoms. We computed a Spearman correlation between the empathy-related NCIs and the positive, negative, and general Positive and Negative Syndrome Scale (PANSS) scores for each time-window throughout the movie. FDR correction was applied to control for the total number of comparisons (131 comparisons) for each of the six main hypotheses (association between ES/ToM NCI and the positive, negative or general symptoms).

### Classification based on ToM and ES NCI

In order to classify participants into their respective groups (Schizophrenia patients and healthy controls) we applied the *k*-nearest neighbor (KNN) classifier, which is a widely used machine learning classification algorithm^33^ based on the closest training examples in the feature space. We used *k* = 10, and classification was carried out in the Matlab Classification Learner application (The MathWorks Inc., Natick, MA, USA). The NCI values per time point per network (simulation and ToM) were used as predictors (total of 302 features). We then assessed generalization through five-fold cross-validation. The overall accuracy, sensitivity, specificity, and positive predictive value (PPV) was computed for each classifier; overall *accuracy* was the number of true classifications to total cohort; *sensitivity* was the number of true positives (i.e. patients correctly classified) for the total number of patients; *specificity* was the number of true negatives (i.e. healthy controls correctly classified) for the total number of healthy controls; *positive predictive value* (PPV) was the ratio of the true positives for all positive classifications (i.e. true and false positives). Statistics were derived from the mean and standard deviation of all 1000 classifiers.

For additional statistical significance assessment of the classification accuracy we conducted a bootstrapping procedure of permutation tests^34^. The class labels of the original dataset were randomly permuted and then classified using the identical aforementioned KNN analysis procedure (using five-fold cross-validation and 1000 repetitions). This procedure was again repeated 1000 times. The *p*-value of the permutation test was defined as the fraction of the number of permuted classifiers that obtained better classification accuracy than the original dataset, given as

$$p = \frac{{{\sum} {\left( {Permuted\;Accuracy \ge Original\,Accuracy} \right)} + 1}}{{k + 1}}$$

where *k* is the number of permutation tests (*k* = 1000).

## Results

### Rating of fear intensity

**Fig. 2:**
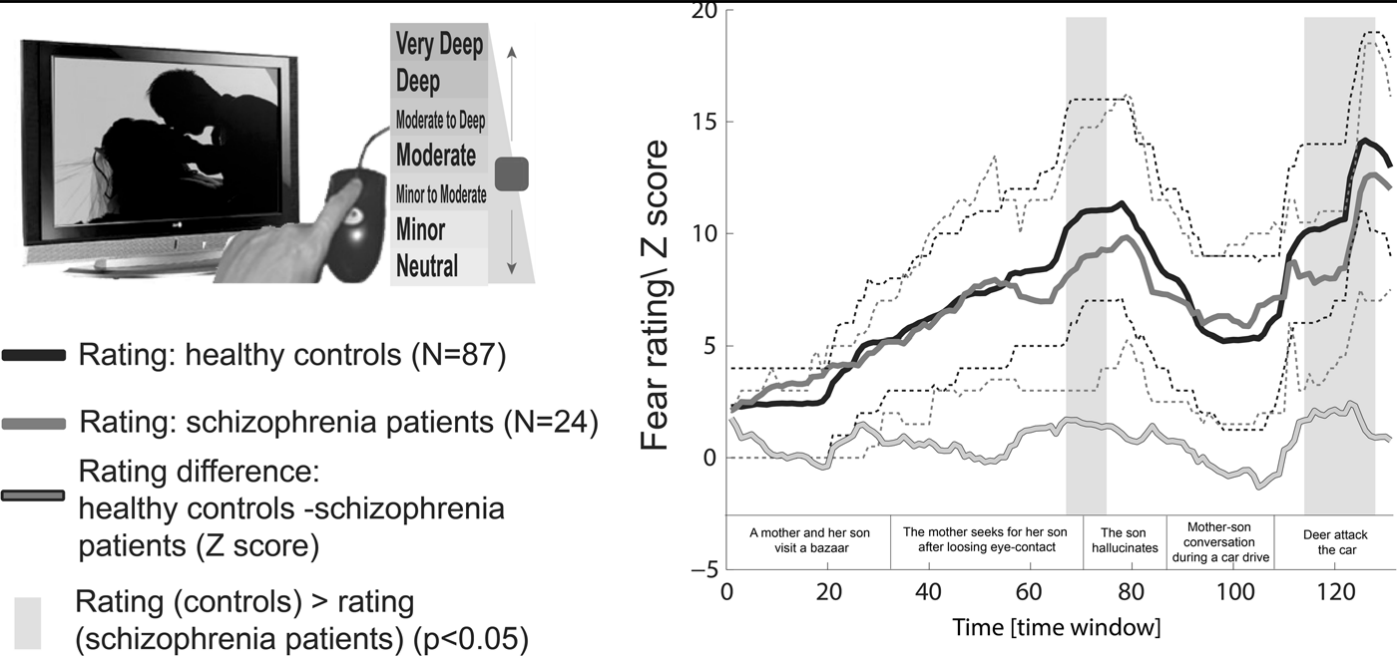
Fear rating by healthy controls and schizophrenia patients for a clip taken from the movie, *The Ring 2* Time courses of moment-to-moment retrospective rating of fear intensity by healthy controls and schizophrenia patients are presented. Solid lines represent the mean rating for each of the groups, whereas dashed lines represent the interquartile range. The time-series of Wilcoxon *Z* scores for the intergroup rating difference is illustrated as a gray curve with black contour. The gray bars indicate time intervals during which the ratings of the groups were different (*p* < 0.05, uncorrected). A brief description of the main events is presented as a time line at the right bottom. The time windows refer to 3-sec shifted windows of 30 seconds each (see Figure S1 for details)

Self-reports obtained from the participants indicated that the cinematic emotional manipulation used in the study was fairly effective. The median peak rating of fear intensity was 16 and 15 out of 21 for the control and patient groups, respectively (corresponding to the titles “high” and “moderate to high” intensity level, respectively; Fig. 2).

We compared the ratings between schizophrenia patients and healthy controls and found no time window in which the intergroup difference was significant at qFDR ≤ 0.05 (i.e., surviving correction for multiple comparisons). However, we located two time intervals in the movie, during which controls rated fear intensity higher than patients at a *p* < 0.05, uncorrected (see Fig. 2). These intervals precede the two global emotional peaks as evident from the median rating of the control group (Fig. 2).

### Cross-correlation analysis of NCI and rating intergroup differences

**Fig. 3:**
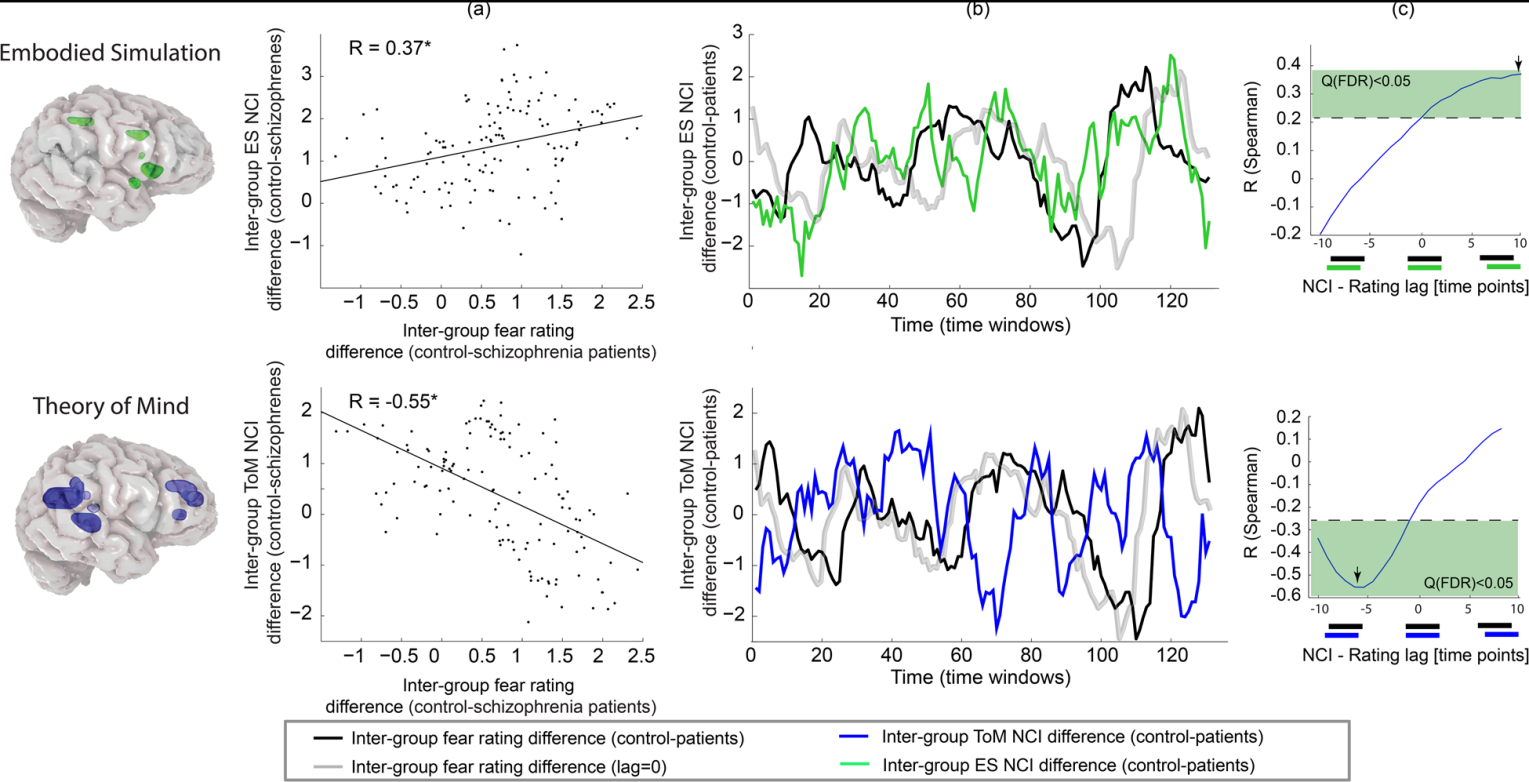
Correlation between intergroup rating and NCI differences **a** Scatter plots of the difference between the fear rating for the healthy controls and schizophrenia patients vs. the corresponding difference between the ES and ToM NCIs (Wilcoxon *z* in both cases). NCI values are taken from a time window that *precedes* the rating time window in 10 TRs in the case of ES, and *succeeds* it in six TRs in the case of ToM. **b** The NCI and rating intergroup differences are presented as time series. To indicate the temporal shift, the gray curve represents the rating difference time series with no lag. **c** Spearman *R* coefficients for cross-correlations between the rating and NCI difference time series. The black arrows indicate the selected time windows that were used in **a** and **b**. The dashed lines indicate a threshold of qFDR ≤ 0.05 for the 21 comparisons in each of the cross-correlation analyses. The time windows refer to 3-sec shifted windows of 30 seconds each (see Figure S1 for details)

To dynamically probe the difference between schizophrenia patients and healthy controls, we used the time series of *Z*-statistics from the Wilcoxon tests comparing fear ratings and NCI for each time window. The intergroup rating difference index was then cross-correlated with the ES and ToM NCI difference indices. These network NCIs showed an opposite pattern of correlation with the rating difference index (Fig. 3). The ES-NCI and the rating difference indices were positively correlated with a peak correlation of 0.37 (qFDR ≤ 0.05) at a lag of 10 (i.e., the *NCI difference preceded the rating difference*; the fact that this peak was indeed a local maximum was confirmed in an additional test of five successive time windows). On the other hand, the ToM-NCI difference index negatively correlated with the rating difference with a minimal Spearman *R* = −0.55 (qFDR ≤ 0.05) at a lag of −6 (i.e., *the NCI difference lagged after the rating difference*). In other words, healthy controls tended to rate their fear higher than schizophrenia patients following an increase in ES connectivity relative to the latter. This increased rating difference was also followed by enhanced ToM connectivity among schizophrenia patients.

### Network cohesion differences between groups

**Fig. 4:**
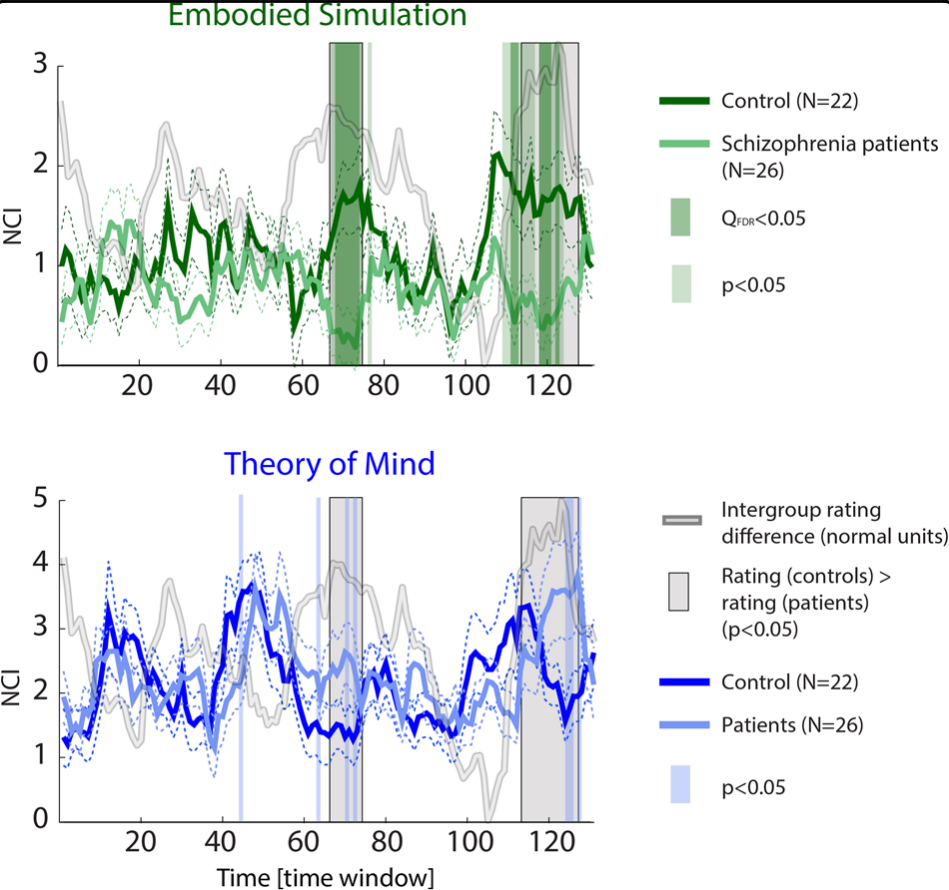
Differences between schizophrenia patients and healthy controls in cohesion of ES and ToM networks during film viewing Time courses of the NCIs computed for the ToM and the ES networks between healthy controls and schizophrenia patients (*N* = 26). Solid lines represent the median values, while the dashed lines indicate the standard error. The colored bars represent time windows during which a significant difference between the NCIs of the groups was evident (*Q*_FDR_ < 0.05, and *p* < 0.05). The Wilcoxon index for intergroup rating (normalized to a 0–5 scale) is overlaid in pale gray. The time windows refer to 3-sec shifted windows of 30 seconds each (see Figure S1 for details)

While the cross-correlation analysis revealed that fluctuations in intergroup NCI differences are functionally meaningful (i.e., related to an emotional measure), it could not indicate specific time points at which patients and controls significantly differed in ES or ToM network connectivity. To directly examine this, the NCI time courses of the ES and the ToM networks were compared between the patient and control groups across each of the 131 time windows of the movie watching condition (Fig. 4).

In the case of ES, we found 12 time windows in which healthy controls showed higher ES NCI (qFDR ≤ 0.05) relative to patients (maximal *Z* = 3.52, *p* < 5 × 10^−4^). Ten of these windows were within the two intervals of maximal intergroup rating difference, as defined by a threshold of *p* < 0.05 (uncorrected). In 11 additional time windows—7 of which were within the maximal rating difference intervals—we found higher ES-NCI at *p* < 0.05, uncorrected. We did not find ToM-NCI intergroup differences at qFDR ≤ 0.05. However, we obtained weaker evidence for higher ToM-NCI in six windows, among schizophrenia at a *p* < 0.05, uncorrected (maximal *Z* = 2.22, *p* < 0.02). Five of these windows were within the maximal rating difference interval. Higher ToM-NCI for the control group was found in one time window at *p* < 0.05.

To examine whether these network differences are specifically associated with connectivity rather than activity level, we compared the time series of averaged activity levels between groups. No significant difference between the groups was found in any of the time windows examined, at either qFDR ≤ 0.05 and *p* < 0.05 (Fig. S2).

### Correlation between NCI and symptom severity

**Fig. 5:**
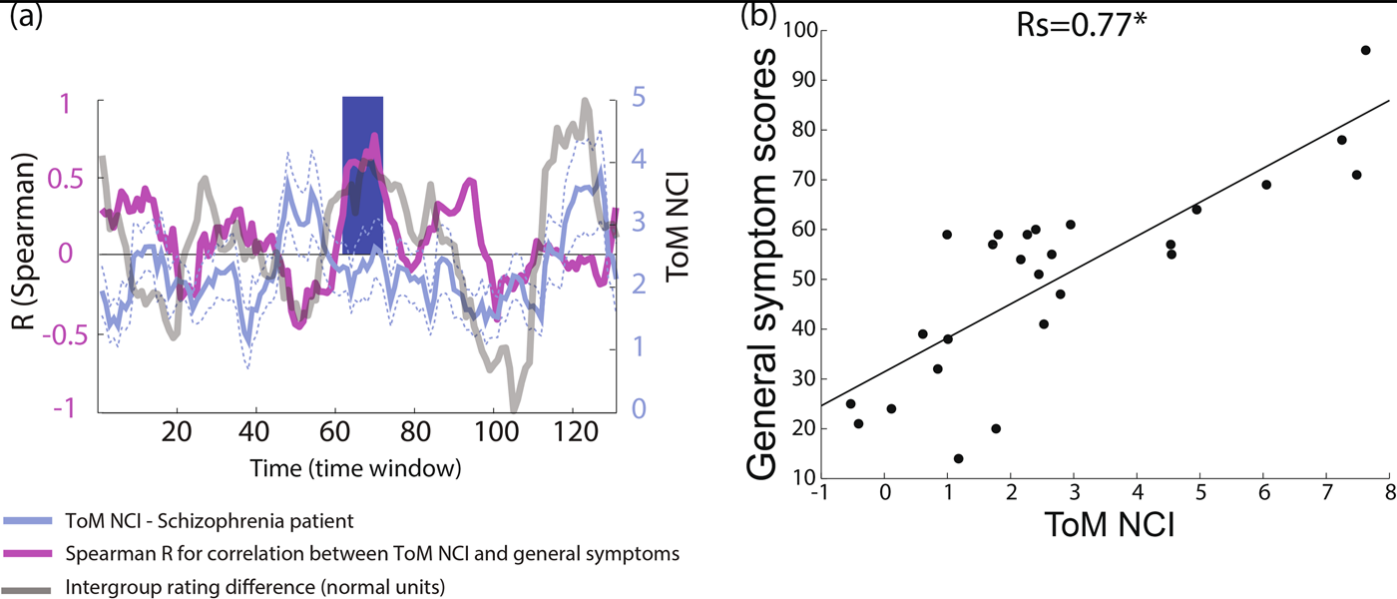
Relationships between schizophrenia general symptoms and ToM NCI **a** Spearman correlation between the general symptoms score and the ToM NCI was computed in every time-window. The resulting time series of the Spearman coefficients is presented in purple along with the median ToM NCI and the standard errors and the intergroup rating difference (see Fig. 4). The blue bar indicates an interval of nine successive time windows in which the correlation survived FDR correction (131 comparisons). **b** General symptom scores plotted vs. individual ToM NCI values in the time window in which a maximal correlation was measured. The time windows refer to 3-sec shifted windows of 30 seconds each (see Figure S1 for details) *qFDR ≤ 0.05, *p* < 5 × 10^−6^

NCI values of our networks of interest were tested for a correlation with negative, positive, and general symptoms. A high correlation (qFDR ≤ 0.05) between the general symptoms and the ToM NCI was found in nine successive time windows included in the first selected interval (Peak *R*s = 0.77, *p* < 5 × 10^−6^, Fig. 5). Five of these windows were within the first maximal intergroup rating difference interval, which corresponds to a cinematic situation in which the mother is anxiously looking for her son. No other correlations were found at qFDR ≤ 0.05 for any other comparison between the symptoms and ToM or ES NCI. However, negative symptoms were also correlated with the ToM NCI at *p* < 0.001 (uncorrected; *R* = 0.62) in the same time window in which the maximal correlation between this NCI and general symptoms was found.

### Classification accuracy and sensitivity

**Fig. 6:**
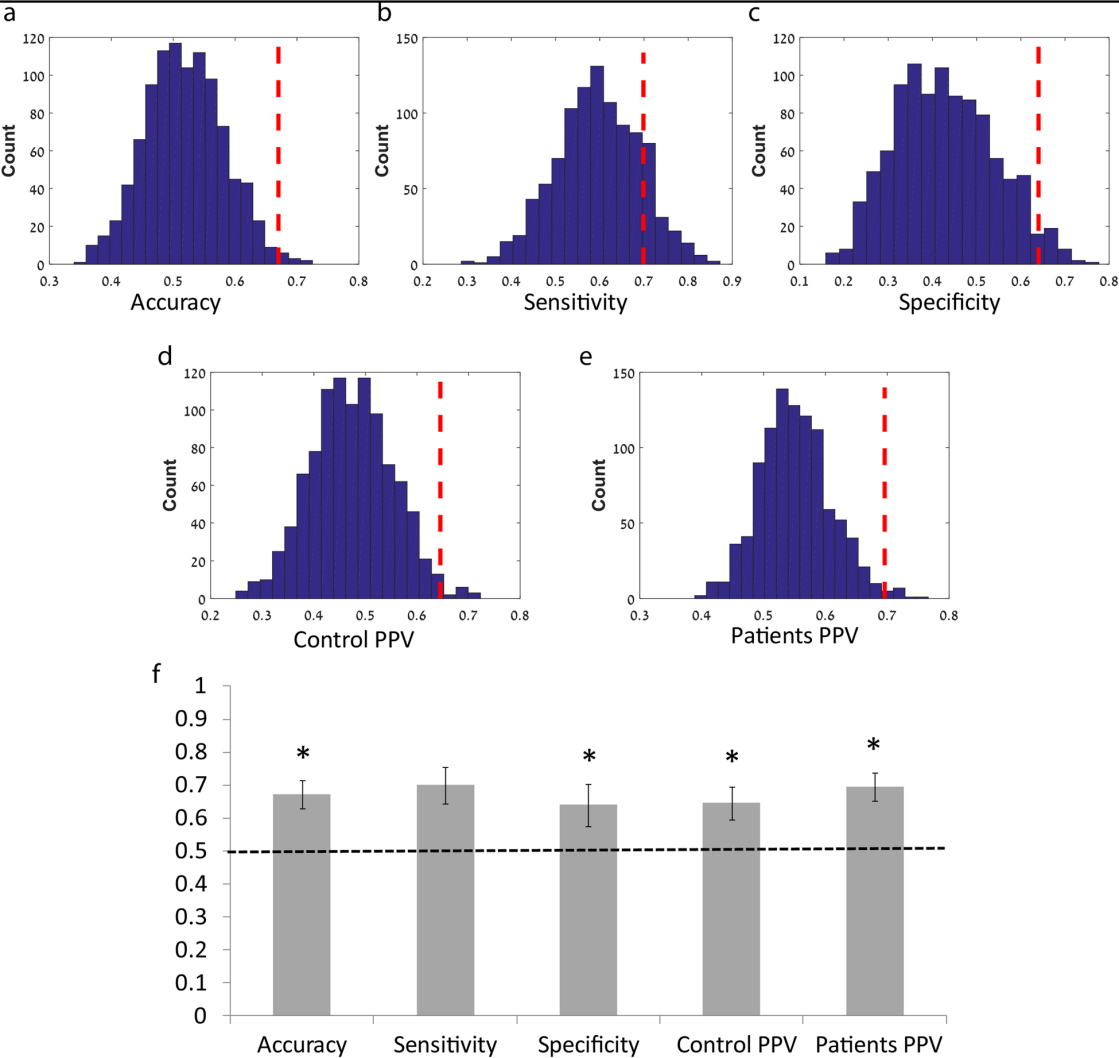
Classification between schizophrenia patients and healthy controls using KNN algorithm In order to discriminate between patients and healthy controls a *k*-nearest neighbor (KNN) machine learning classification algorithm was conducted on the NCI results (five-fold cross-validation and 1000 repetitions). For statistical inference the analysis, a bootstrapping procedure of permutation tests was conducted 1000 times on random permutations of the dataset for the classification overall accuracy **a**, sensitivity **b,** specificity **c**, and positive predictive value (PPV) of healthy controls **d**, and schizophrenia patients **e**. The dashed red vertical lines represent the classification accuracy of the original dataset. The bar graph demonstrates the classification accuracy features for the KNN classifier **f**. The dashed line denotes the chance level accuracy of 50% and the error bars represent the standard deviation calculated on 1000 repetitions. This analysis revealed significant classification power with overall accuracy of 67% (*p* = 0.012), with sensitivity of 70% (*p* = 0.15) and specificity of 64% (*p* = 0.035). The positive predictive value (PPV) were found significant both for the HC group (63%, *p* = 0.014) and for the schizophrenia group (69%, *p* = 0.015)

The machine learning KNN algorithm revealed significant classification power (Fig. 6). The overall accuracy was 67% (0.67 ± 0.042, *p* = 0.012), with a sensitivity of 70% (0.70 ± 0.054, *p* = 0.15) and specificity of 64% (0.64 ± 0.061, *p* = 0.035). The positive predictive values for the healthy controls were 63% (0.63 ± 0.067, *p* = 0.014) and 69% (0.69 ± 0.057, *p* = 0.015) for the Schizophrenia group.

## Discussion

Applying a novel tool for analyzing network cohesion dynamics and its association with the evolving emotional experience during fearful film-clip viewing, revealed specific abnormalities in the time-sensitive functional connectivity of networks related to social cognition among schizophrenia patients, as compared to healthy controls. While schizophrenia patients and healthy controls showed similar patterns of continuous fear rating, the healthy controls displayed a tendency to react to the cinematic threat sooner, both in terms of conscious feelings and changes in network cohesion (Figs. 2 and 3). The cross-correlation analysis showed that among healthy controls the earlier increase in reported fear intensity at peak cinematic periods was preceded by a greater increase in the cohesion index of the ES network, and was followed by a greater decrease in ToM network cohesion (Fig. 3). The healthy controls also showed the expected pattern of a robust increase in ES cohesion (ES-NCI) and a simultaneous modest decrease in ToM-NCI during the emotional peaks (Fig. 4), while schizophrenia patients showed an opposite pattern; decreased ES-NCI and a modest increase in ToM-NCI. The group differences were found to be more robust for ES-NCI. Notably, this difference was found when comparing NCIs rather than BOLD levels of activity (Fig. S2), demonstrating that our network cohesion dynamics may indeed reveal meaningful variability that is overlooked by standard BOLD analyses of specific regions. Classification analysis importantly suggests that context related ToM-NCI and ES-NCI dynamics have the potential to serve as a psychopathology marker, although this notion must be further validated for disorder specificity. However, further support of this potential use is also evident in the emotional peak related ToM-NCI enhancements and their correlation with symptom severity among patients.

### Abnormal ES network cohesion

Increased ES network cohesion among healthy subjects during fearful cinematic peaks may reflect the enhanced simulation of the cinematic character’s bodily reactions to threat^15,35^. Interestingly, the schizophrenia patients showed an opposite pattern of decreased ES-NCI during these same cinematic moments. However, this group difference was not reflected in activation patterns, suggesting a particular effect in the network dynamic metric of cohesion as depicted by our NCI analysis. Importantly, the difference in ES-NCI was preceded by a slight difference in the fear ratings between the groups (Fig. 3). Our analysis revealed a difference in the dynamic inter-relations between neural and behavioral responses of the two groups, alluding to the importance of temporal variation in the formation of the subjective emotional experience^36^. Our findings are in line with the notion that schizophrenia patients have a breakdown in the automatic embodiment of social cues^37,38^. This is also consistent with the accumulating evidence regarding anatomical and functional abnormalities in major ES nodes among such patients. These abnormalities have been reported mainly in the anterior insula and the dorsal ACC, including decreased activation during emotional conditions^8^, reduced resting state functional connectivity^39^ and cortical volume^40^. In fact, volume abnormality in these two regions has recently been suggested in a transdiagnostic common ground perspective in psychiatry^41^.

It should be noted that the ES network nodes considerably overlap with the “salience” network, which has been found during resting states and associated with the processing of acute homeostasis-changing cues^42^. The results presented here are also in line with Kapur’s theory^43^, postulating that schizophrenia involves a deficiency in assigning saliency to the actual experience, which may be a result of a dysregulated hyper-dopaminergic state. Our findings of decreased cohesion in the ES network during peak moments of cinematic threat and differences in enhanced ES network cohesion prior to increased fear ratings (Figs. 3 and 4), expand upon this suggested deficiency, likely indicating a deficit in the processing of social-affective saliency, known to be critical for appropriate social somatic affiliation. It is important to state that our analysis revealed that this difference in enhanced ES network cohesion emerges during specific time points in relation to the emotional dynamics. Thus, the deficiency in ES network connectivity among schizophrenia patients may be context-dependent as opposed to a constant tonic feature. The fact that we found no significant correlation between ES-NCI and schizophrenia could be due to the poor resolution of symptom categories. This may point to an underestimation of the processing deficiencies of salience-related social content.

### Abnormal ToM network cohesion

Multiple studies of social cognition among schizophrenia patients have consistently demonstrated large effects sizes for ToM deficiencies^7,35,44,45^. In terms of brain activity, a recent meta-analysis of schizophrenia-related abnormalities during ToM tasks^10^ revealed a complex pattern within the related network: while mPFC and a section of the left TPJ were found to be under-activated in schizophrenia patients, bilateral over-activation of the more dorsal aspect of the TPJ were also associated with the pathology. Superficially, our findings of increased ToM-NCI during peak emotions, are seemingly at odds with this meta-analytical picture, since the opposite direction of the signals within the ToM network is expected to manifest as reduced network cohesion. However, considering that we applied dynamic connectivity measures of the network, the findings seem to be complementary rather than contradictory.

Importantly, while previous studies investigated ToM deficiencies during the performance of specific mentalization tasks, our study examines the connectivity dynamics of a ToM-related network in the absence of an explicit mentalization task. In fact, the largest difference between patients and controls emerged during dramatic peaks when healthy subjects tended to reduce ToM-NCI. The reduction of ToM network cohesion among healthy controls, when facing an immediate and salient cinematic event, may be associated with the deactivation of the default mode network (DMN) during enhanced processing of external stimuli. Indeed, major nodes of the DMN, such as the mPFC, PCC, lateral temporal cortex, and the angular gyrus are all part of the ToM network^46^. In fact, several functional and anatomical DMN neuroimaging abnormalities have been reported in schizophrenia (for a review, see ref. ^38^). While most of these functional abnormalities were found during resting state (e.g. ^39^), a line of studies have shown reduced DMN deactivation in schizophrenia patients during working memory^39–41^ and verbal tasks^47,48^; during which healthy controls consistently showed DMN suppression. More so, abnormality in DMN was evident not only in terms of its activation pattern but also in terms of functional connectivity, mostly pointing to a hyper-connectivity^39,49^.

In light of this set of evidence, it is possible that the pattern of hyper-ToM network cohesion, in association with a growing emotional load during movie viewing among schizophrenia patient, reflects a pathological reaction pattern to externally driven salient social events. The finding that fearful moments in the movie, which call for here-and-now relatedness to the environment, are followed by an increase in ToM-NCI among schizophrenia patients is in line with the findings of reduced DMN deactivation during certain tasks and/or enhanced resting state functional connectivity; reflecting greater internally generated processing, such as mind-wandering. Moreover, the DMN is strongly implicated in mind wandering and in autobiographical planning and internally guided thoughts^2^. Specifically, activity in the core DMN nodes is positively related to mind wandering as indicated by introspective thought sampling and attentional lapses in the form of behavioral errors^50^. Indeed, schizophrenia patients exhibited a higher frequency of mind wandering relative to healthy controls, and this was correlated with symptom severity. The authors suggest that such elevated internally generated processing might explain the reported feeling of “detachment from reality” in schizophrenia^49^.

Taken together, these findings point to the intriguing possibility that schizophrenia patients tend to react to salient emotional cues about others not only with reduced arousal, but also with active switching from the processing of externally generated to internally generated information. The possibility that this reaction pattern is pathological is supported by the correlation found between ToM-NCI and general schizophrenia symptoms during one of the emotional peaks (Fig. 5).

### Abnormal balance between ES and ToM networks

An intriguing aspect of our work is the reversed balance between ES and ToM networks as they relate to an increasing emotional experience, such as viewing the distress of another person. Reorganization of large-scale brain networks when under extreme emotional conditions has been suggested to be crucial for flexible and adaptive behavior^51^. Accordingly, the abnormal connectivity dynamics found for social-affective-related network cohesion, as associated with reported changes in cinematic fear, may reflect a deficient allocation of relevant resources for the facilitation of adaptive social cognition and emotions among schizophrenia patients. Although speculative, it is possible that the imbalance between the dominance of ToM-NCI and ES-NCI during threatening moments contributes to the known stress vulnerability in schizophrenia, often triggering the onset of psychotic episodes or recurrent exacerbations^13^. The clinical significance of this network imbalance is further supported by the predictive classification analysis derived from the network cohesion measurements (Fig. 6), although further work should be pursued to demonstrate that such a prediction is indeed disorder specific.

### Limitations

Our study presents some limitations. The lack of a controlled measure of social cognition hampers the validity of our results in terms of this process domain. Yet, the reliance on a priori functionally defined network (rather than resting state networks) supports our assumptions regarding social cognition processing.

In addition, although most of the patients were in their first psychotic episode, many had already received antipsychotic medication, albeit for a short period of time (mostly few months). Although we cannot rule out the possibility that our findings reflect the drug effects, we believe that it is more likely that the medications would contribute to tonic affective deficiencies rather than to context-specific influences (the patients showed a pattern of increasing ToM and decreasing ES NCI in adjacency to emotional peaks). More so, our findings are congruent with previous studies that controlled for the drug effects. Resting-state functional connectivity have consistently found abnormal functional connectivity within the DMN among schizophrenia patients, including medication-naive subjects^52,53^. Abnormal functional connectivity of the DMN has also been reported among unmedicated first-degree relatives, as compared to healthy controls, during a working memory task^54^.

Lastly, another potential caveat is the uncertainty regarding the weight of social aspects in our cinematic fearful stimulus. It may be the case that horror elements, such as darkness and soundtrack manipulations elicit fear regardless of processes of social cognition that take place during the film viewing. Our assumption that this movie elicits fear that involves processes of social cognition is based on the theory of the film researcher Ed Tan^22^. In his account of cinematic emotions, Tan defines “empathic emotions” as emotions that are elicited in relation to the concerns of the cinematic characters rather than to the concerns of the viewer herself. Thus, empathic fear is experienced when the characters’ rather than the viewer’s life is put under threat. In the clip from The Ring 2 the characters address threats to their own well-being: the mother is looking for her lost boy, and both of them are later violently attacked. However, it is yet to be examined if the effect we observed in our study could be replicated when no human character mediates the fear. In this case, it could be that the saliency of the event, rather than its social aspects, triggers the opposing network connectivity patterns.

In conclusion, using a naturalistic empathically distressful cinematic stimulus along with novel network cohesion dynamic analysis, this fMRI study suggest that schizophrenia implicates the abnormal dynamics of networks related to social-affective processing. The known overlap between ES and ToM networks with intrinsic networks involved in salience and mentalization processing a provides broader explanation of our findings. The decreased ES cohesion among schizophrenia patients, prior to peak differences in emotional experiences, indicates a neural source for false attribution to externally driven socially salient cues. Although speculative, the finding of the abnormal balance between ES and ToM networks when facing the distress of others might underlie the tendency of schizophrenia patients to detach from social salient cues while engaging in internally-generated active mentalization. The significant classification based on ES and ToM NCIs, as well as the correlation between enhanced ToM-NCI and symptom severity further support such a proposition. Although, further investigation with larger a sample size and specific social cognition measures is needed.

## Electronic supplementary material

Table S1

Table S2

Supplementary Information

Figure S1

Figure S2
