# Supplementary material for: Social affective context reveals altered network dynamics in schizophrenia patients
Source: Transl Psychiatry. 2018 Jan 31;8:29. doi: 10.1038/s41398-017-0055-9 (PMC5802465; doi:10.1038/s41398-017-0055-9)
Supplement: Supplementary file 1 — Table S1 [file 41398_2017_55_MOESM1_ESM.docx]

**Table S1: Demographic details and symptoms**

**Schizophrenia patients**

|  | Hospital | gender | Mother Language | Education | Age | Diagnosed | Medications | PANSS total | PANSS positive | PANSS Negative | PANSS general | CGI |
| --- | --- | --- | --- | --- | --- | --- | --- | --- | --- | --- | --- | --- |
| 1 | Mazra | M | ARB/HEB | 12 | 19 | 3 months | , dekinet 2mg, modikat Serequal, | 86 | 17 | 30 | 39 | 5 |
| 2 | Mazra | M | ARB/HEB? | 12 | 21 | 2 months | halidol, serequal, dekinet 2 mg | 83 | 17 | 28 | 38 | 4 |
| 3 | Mazra | F | HEB | 12 | 30 | 5.5 months | zyprexa - 2 in a day, clopixol dipo, diralin | 52 | 14 | 14 | 24 | 4 |
| 4 | Mazra | M | ARB/HAB | 7 | 36 | 5 months | sm clopexol dipo - injection once in 2 weeks | 48 | 13 | 15 | 20 | 5 |
| 5 | Mazra | M | HEB | 12 | 24 | 1.5 months | zyprexa | 67 | 18 | 17 | 32 | 4 |
| 6 | Mazra | M | HEB | 12 | 20 | 2.5 months | Serequal, clonex | 147 | 33 | 36 | 78 | 5 |
| 7 | Mazra | F | HEB | 12 | 29 | 2 months | Clopexol, risperidal 4mg/d | 134 | 34 | 29 | 71 | 5 |
| 8 | Mazra | M | HEB/ARB | 10 | 28 | 30 months | Risperdal 2mg/d, zyprexa 10 mg/d, clonex | 167 | 38 | 39 | 90 | 5 |
| 9 | Beer-yaakov | M | HEB | 12 | 23 | 5 months | risperdal 2mg/d, zyprexa | 98 | 22 | 29 | 47 | 4 |
| 10 | Beer-yaakov | F | HEB | 12 | 24 | 3 months | risperdal 4mg/d | 136 | 25 | 42 | 69 | 6 |
| 11 | Beer-yaakov | M | HEB/Russion | 14 | 25 | 2 months | risperdal 4mg/d, clonex 0.5mg*2/d | 104 | 23 | 26 | 55 | 4 |
| 12 | Beer-yaakov | M | HEB | 11 | 26 | 6 months | risperdal 4mg/d | 115 | 28 | 31 | 54 | 5 |
| 13 | Beer-yaakov | F | HEB/ARB | 18 | 30 | up to 2 years | risperdal 4mg/d, clonex 1mg/d | 110 | 27 | 24 | 59 | 5 |
| 14 | Beer-yaakov | F | HEB/English | 12 | 28 | 2 months | i.m risperdal 25mg/2h, rispond 2mg/d, vaben 10mg*3/d | 116 | 25 | 32 | 59 | 5 |
| 15 | Beer-yaakov | M | HEB | 12 | 23 | few months | risperdal 2mg/d | 104 | 25 | 28 | 51 | 4 |
| 16 | Beer-yaakov | F | HEB/Amharic | 12 | 26 | 2 months | Risperdal 4mg/d | 116 | 25 | 30 | 61 | 5 |
| 17 | Beer-yaakov | M | HEB/Russian | 12 | 20 | few months | perphenan 24mg/d, lorivan 1mg/d | 125 | 33 | 32 | 60 | 5 |
| 18 | Beer-yaakov | F | HEB | 12 | 24 | 8 months | risperdal 3mg/d, clonex 0.5mg/d | 114 | 21 | 29 | 64 | 4 |
| 19 | Beer-yaakov | F | HEB | 7 | 22 | 2 months | Risperdal 4mg/d | 123 | 27 | 37 | 59 | 5 |
| 20 | Beer-yaakov | F | HEB/Russian | 12 | 18 | 3 months | risperdal 4mg/d, dekinet 2mg/d | 105 | 28 | 22 | 55 | 4 |
| 21 | Beer-yaakov | M | HEB/Russian | 12 | 25 | unknown | risperdal 2mg/d | 114 | 28 | 29 | 57 | 4 |
| 22 | Beer-yaakov | M | HEB/Amharic | 10 | 19 | unknown | zyprexa 20mg/d | 90 | 15 | 34 | 41 | 3 |
| 23 | Beer-yaakov | M | HEB/Russian | 12 | 29 | 4 months | zyprexa 10 mg/d, clonex | 111 | 22 | 32 | 57 | 4 |
| 24 | Beer-yaakov | F | HEB | 12 | 30 | 6 weeks | Risperdal 2mg/d | 31 | 7 | 7 | 14 | 3 |
| 25 | Lev-hasharon | M | HEB | 9 | 40 | 1 year | saphris 20mg/d, assival, 15mg/d, dekinet 2mg/d, nocturno 7.5mg/d | 60 | 30 | 9 | 21 | 4 |
| 26 | Lev-hasharon | M | HEB/Russian | 12 | 25 | 2 months | risperdal 4mg/d clonex 0.5mg/d | 46 | 12 | 9 | 25 | 3 |
| 27 | Lev-hasharon | M | HEB/Amharic | 12 | 26 | 3 months | assival 10mg, dekinet 2mg, risperdal 2mg | 70 | 22 | 18 | 30 | 4 |

**Healthy controls**

|  | **gender** | **Mother Language** | **Education** | **Age** |
| --- | --- | --- | --- | --- |
| 1 | M | HEB/English | 18 | 34 |
| 2 | F | HEB/English | 15 | 25 |
| 3 | F | HEB/English | 15 | 25 |
| 4 | M | HEB/English | 16 | 28 |
| 5 | M | HEB/English | 16 | 35 |
| 6 | M | HEB | 12 | 23 |
| 7 | M | HEB/English | 17 | 28 |
| 8 | F | HEB/English | 12 | 23 |
| 9 | F | HEB/English | 12 | 22 |
| 10 | F | HEB/English | 16 | 26 |
| 11 | F | HEB/English | 16 | 31 |
| 12 | F | HEB/English | 17 | 33 |
| 13 | F | HEB/English | 12 | 30 |
| 14 | M | HEB/English | 17 | 35 |
| 15 | F | HEB/English | 16 | 28 |
| 16 | F | HEB/English | 15 | 27 |
| 17 | F | HEB/English | 17 | 26 |
| 18 | M | HEB/English | 16 | 27 |
| 19 | M | HEB | 12 | 26 |
| 20 | M | HEB/English | 16 | 39 |
| 21 | F | HEB/English | 12 | 25 |
| 22 | M | HEB/English | 12 | 22 |
