# Supplementary material for: Social affective context reveals altered network dynamics in schizophrenia patients
Source: Transl Psychiatry. 2018 Jan 31;8:29. doi: 10.1038/s41398-017-0055-9 (PMC5802465; doi:10.1038/s41398-017-0055-9)
Supplement: Supplementary file 2 — Table S2 [file 41398_2017_55_MOESM2_ESM.docx]

**Table S2: Network nodes and the Talairach coordinates of their centers**

| Region | x | y | z |
| --- | --- | --- | --- |
| ToM network | | | |
| Ventromedial prefrontal cortex | 3 | 50 | 2 |
| Dorsomedial prefrontal cortex | 4 | 54 | 26 |
| Right temporoparietal junction | 49 | -54 | 24 |
| Left temporoparietal junction | -58 | -48 | 19 |
| Right superior temporal sulcus | 54 | -41 | 5 |
| Left superior temporal sulcus | -54 | -33 | 2 |
| Precuneus | -2 | -51 | 31 |
| ES network | | | |
| Medial/anterior cingulate cortex | 2 | 14 | 34 |
| Right anterior insula (dorsral) | 38 | 24 | 9 |
| Left anterior insula\ inferior frontal gyrus | -41 | 14 | 2 |
| Left anterior insula (ventral) | -31 | 4 | -11 |
| Precuneus/paracentral lobule | 7 | -45 | 50 |
