# Supplementary figures and images for: Social affective context reveals altered network dynamics in schizophrenia patients
Source: Transl Psychiatry. 2018 Jan 31;8:29. doi: 10.1038/s41398-017-0055-9 (PMC5802465; doi:10.1038/s41398-017-0055-9)

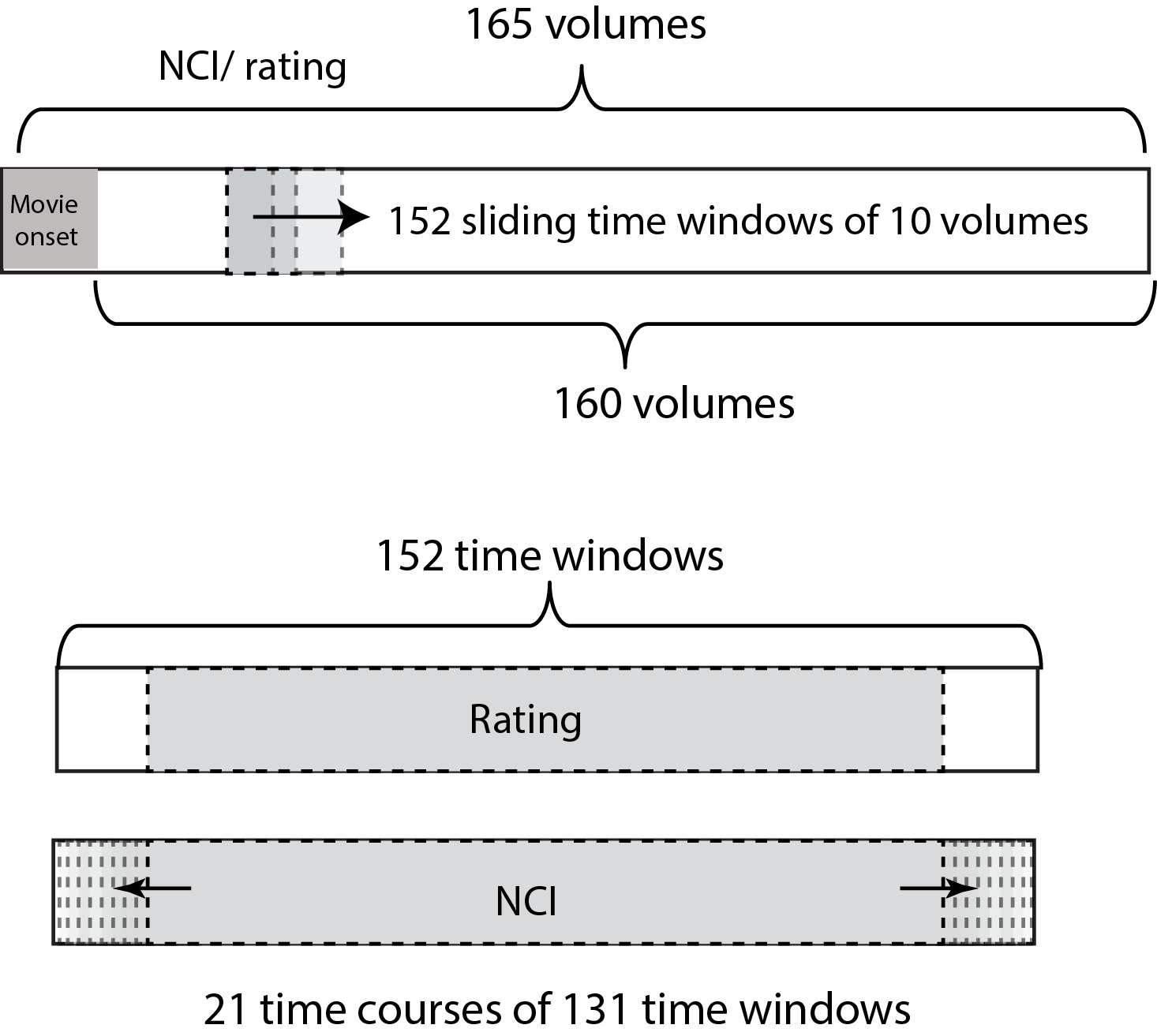

Supplement: Supplementary file 4 — Figure S1 [file 41398_2017_55_MOESM4_ESM.tif]

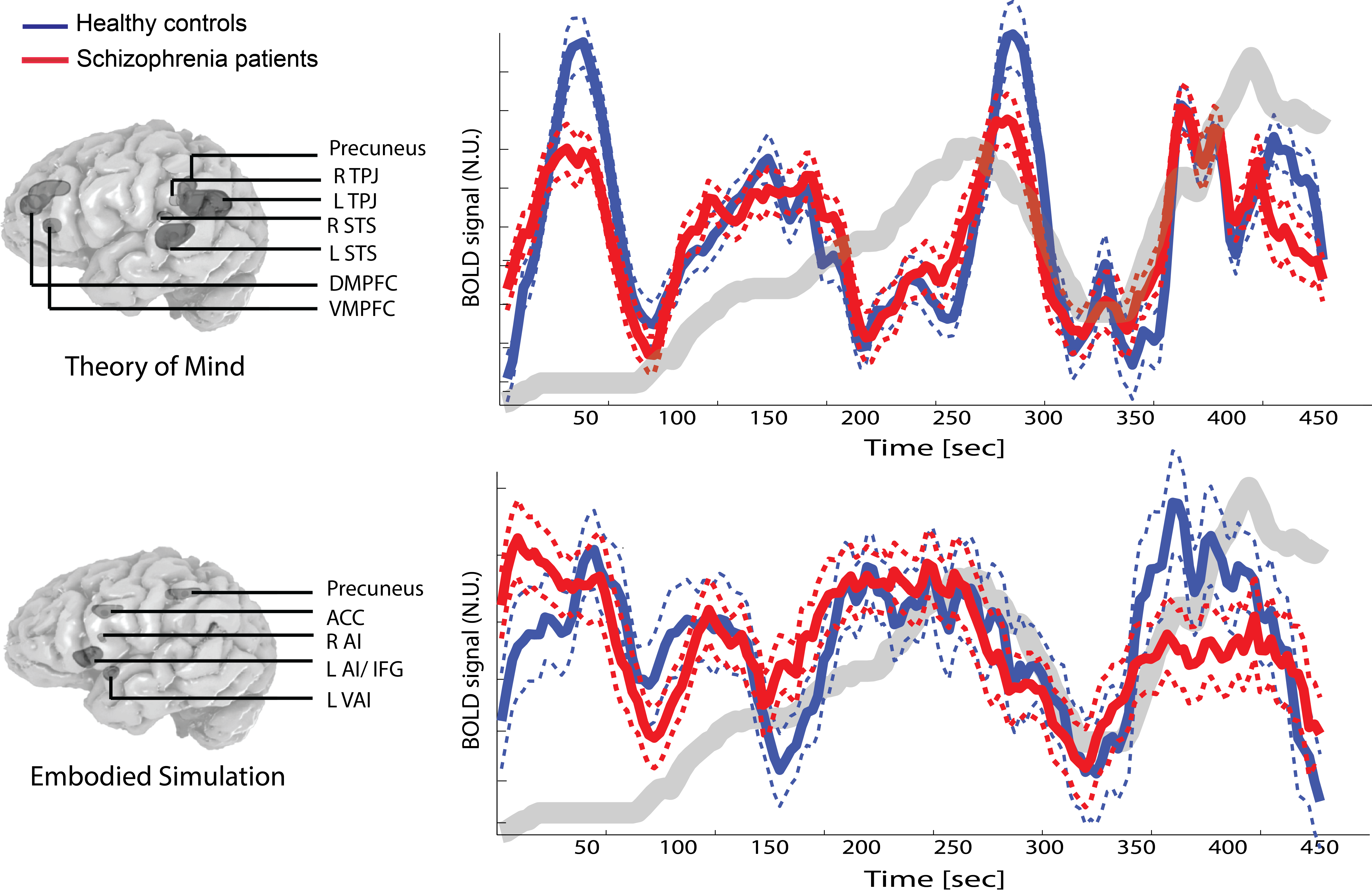

Supplement: Supplementary file 5 — Figure S2 [file 41398_2017_55_MOESM5_ESM.tif]
